# Berberine in Combination with Insulin Has Additive Effects on Titanium Implants Osseointegration in Diabetes Mellitus Rats

**DOI:** 10.1155/2015/824259

**Published:** 2015-12-13

**Authors:** Li Lu, Huang Zhijian, Li Lei, Chen Wenchuan, Zhu Zhimin

**Affiliations:** Department of Prosthodontics, West China Hospital of Stomatology, State Key Laboratory of Oral Diseases, Sichuan University, Chengdu 610041, China

## Abstract

This study evaluated the effects of berberine in combination with insulin on early osseointegration of implants in diabetic rats. Fifty male Sprague-Dawley rats were randomly divided into 5 groups: healthy rats were used as control (HC), and streptozotocin-induced diabetic rats were treated with insulin, berberine, berberine + insulin (IB), or no treatment. Each rat received one machined-surface cp-Ti implant into the right tibia and was given insulin injection and/or gavage feeding with berberine daily for 8 weeks until being sacrificed. Serum levels of alkaline phosphatase (ALP) and bone gamma-carboxyglutamic acid-containing protein (BGP) were analyzed in each group. Peri-implant mineral apposition was marked by fluorochrome double-labeling and osseointegration was histomorphologically examined. The ALP and BGP levels decreased in diabetic rats but were successfully corrected by insulin and berberine combined treatment. Moreover, untreated diabetic rats had less labeled mineral apposition and impaired osseointegration. In contrast, Groups I, B, and IB were observed with increased peri-implant bone formation. The combination treatment of insulin and berberine was more effective than each administrated as a monotherapy. These results suggest that berberine combined with insulin could promote osseointegration in diabetic rats, thereby highlighting its potential application to patients, though further studies are needed.

## 1. Introduction

“Osseointegration” refers to the formation process of a direct interface between an implant and living bone [1]. Thereafter, implant has been extensively studied and widely used in clinics to replace missing teeth and restore oral functions. However, many systemic diseases such as osteoporosis, diabetes, and autoimmune diseases may interfere with the implant osseointegration in clinical practice [2]. Studies have shown that diabetes caused a higher failure rate of implants and poorer bone implant integration [3, 4]. Type 2 diabetes mellitus (T2DM), the most common form of diabetes mellitus, is a metabolic disorder characterized by hyperglycemia resulting from peripheral insulin resistance with defective insulin secretion. T2DM is also associated with systemic chronic progressive disorders in kidneys, nerves, blood vessels, bones, and other tissues, which can lead to diminished immune response and increased inflammatory effect [5, 6]. Also, substances such as advanced glycation end products (AGEs) and cytokines have deleterious effects on osteoblasts and the bone marrow microenvironment [7]. All of the systemic changes above would impair the osseointegration, as well as the long-term function of dental implants.

It is accepted that survival of implant can be improved when plasma glucose level is brought under control [8]. However, others admit that insulin alone is insufficient to reverse all the negative impact of diabetes on bone healing, with impaired bone implant integration in both animal models and patients [9–11]. One explanation may be the impossibility of continuously monitoring glucose and automatically adjusting insulin delivery; another important reason may lie in insulin resistance. Hence, there is a considerable interest in seeking complementary and alternative approaches in improving implant success in diabetes, especially orally available drugs which could mimic insulin action and somehow overcome insulin resistance [12].

Berberine (BBR, C_20_H_18_NO_4_) is an isoquinoline alkaloid purified from herbal plants including* Coptis chinensis*,* Hydrastis canadensis*, and* Berberis aquifolium* [13]. This naturally occurring molecule displays a broad array of pharmacological effects beneficial to a number of diseases. For example, it has been used as a nonprescription drug for treating infectious diseases like diarrhea in Asia for centuries. The hypoglycemic effect of berberine was first discovered in 1988 by accident when diabetic patients were found to have lowered serum glucose level with berberine playing its antidiarrheal role [14]. Thereafter, more studies have shown that berberine could potently augment glucose uptake into adipose and muscle tissues through multiple mechanisms, including insulin-dependent PTP1B, insulin-independent AMPK, increasing the affinity of low-activity glucose transporters (gluT1), and improving insulin sensitivity [15–17]. In STZ-induced diabetic rats, berberine potently lowered rats fasting blood glucose levels and improved oral glucose tolerance. Additionally, berberine also lowered multiple factors related to insulin resistance including blood cholesterol and triacylglycerols levels [18]. In diabetic patients, the hypoglycemic effect of berberine was similar to that of metformin [19]. In another aspect, berberine has been reported to promote osteogenic differentiation of bone marrow-derived mesenchymal stem cells (MSCs) through canonical Wnt/*β*-catenin signaling [20]. Also, osteogenic genes expression, including* osteopontin*,* osteocalcin*, and* Runx2* (Runt-related transcription factor 2), was upregulated in osteoblasts by berberine [21]. Furthermore, berberine inhibited osteoclasts activity through suppressing the NF-kappaB and Akt pathways [22, 23]. In vivo, osteoporosis in both glucocorticoid-induced rat model and senescence accelerated mice P6 (SAMP6) was prevented by berberine [24, 25]. Since functions of berberine in glucose homeostasis, insulin sensitizing, and bone anabolism are closely related to the promotion of bone formation in diabetic patients, we therefore hypothesize that berberine alone and in combination with insulin therapy may be beneficial to the implant osseointegration in T2DM. The combination therapy seems to be more promising for the different and complementary mechanisms of action of berberin and insulin.

## 2. Experimental Section

### 2.1. Experimental Implants

The implants were provided by Professor Liu Xiaoguang (the National Engineering Research Center of Biomaterials, Sichuan University, Chengdu, China). The cp-Ti implants are 4 mm in length and 1 mm in diameter with machined surface. All implants were washed with deionized water in the ultrasonic bath and sterilized in an autoclave before surgery.

### 2.2. Experimental Animals

This experiment protocol was approved by the Animal Research Bioethics Committee of Sichuan University and was conducted following the international animal welfare standards. A total of 50 male Sprague-Dawley (SD) rats, provided by the Experimental Animal Center at Sichuan University, were selected for study. They were 8 weeks of age, weighing around 190 g. Every two rats were housed in each cage in a 12-hour day/night cycle and had free access to water and rat normal pellet diet (NPD, DaShuo Laboratory Animal Co., Ltd., Chengdu, China).

After 1 week of acclimation (start of the study), the rats were randomly assigned into 5 groups (*n* = 10 per group): (1) healthy rats without treatment (Group HC); (2) diabetic rats without treatment (Group D); (3) diabetic rats treated with insulin (Group I); (4) diabetic rats treated with berberine (Group B); (5) diabetic rats treated with both insulin and berberine (Group IB). Type 2 diabetes rat model was built according to the combination of high-fat diet-fed and low-dose streptozotocin-treatment method [26]. Four diabetic groups were fed with high-fat diet (HFD, 58% total kcal of fat, 25% of protein, and 17% of carbohydrate) while Group HC were continually fed with NPD. After 2 weeks of dietary manipulation, the high-fat diet rats (i.e., Groups D, I, B, and IB) were given intraperitoneally 35 mg/kg freshly prepared streptozotocin (STZ, Sigma, St. Louis, MO, USA), while the healthy control rats (Group HC) were injected with 1 mL of vehicle citrate buffer (pH 4.4). The plasma glucose levels (PGLs) were measured 72 hours after the STZ administration and rats with nonfasting PGLs higher than 16.7 mmol/L threshold were considered to be diabetic and chosen for further studies. All the rats were continually fed on their respective diets until being sacrificed.

### 2.3. Implant Surgery

**Figure 1:**
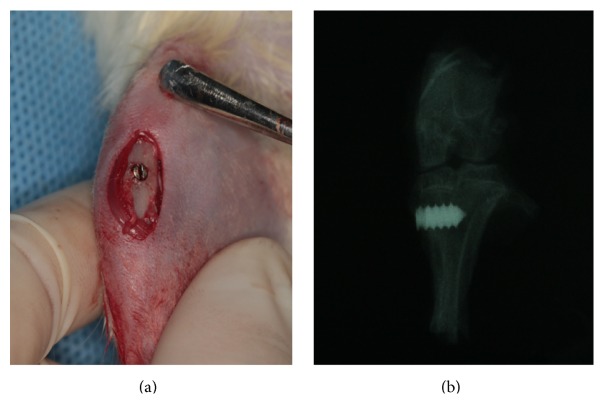
Implant surgery and postsurgery radiograph. (a) The implant was placed into the hole prepared in the tibia metaphysis. (b) Radiograph of tibia with implant after surgery.

After diabetic rat model establishment (0 days), animals were anesthetized intraperitoneally (10% chloral hydrate, 3 mL/kg). The surgical area was shaved and disinfected with 75% ethanol. Then, the implant bed was prepared by standardized drilling procedure with saline irrigation [27], followed by inserting the implant perpendicularly to the long axis of the right tibia in distal tibia metaphysis (2 mm distal to the proximal growth plate) (Figure 1). After surgery, the skins were carefully and separately sutured to ensure the submerged healing of implants with no functional loading. All animals received the intramuscular antibiotic prophylaxis in the first three days after surgery.

### 2.4. Pharmaceutical Treatment

**Figure 2:**
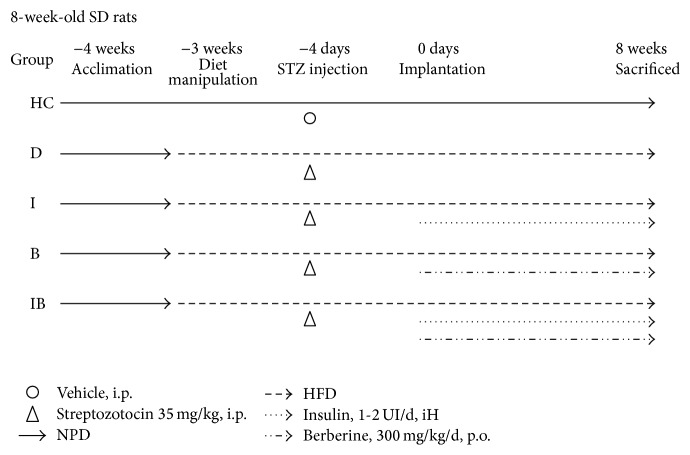
Summary of animal grouping and treatments. Animals were randomly allocated into five groups: healthy rats as control (HC) and 4 groups of diabetic rats including (1) no additional treatment, (2) insulin, (3) berberine, and (4) berberine + insulin therapies; the treatments were given to animals for 8 weeks until being sacrificed.

Group I rats received a daily subcutaneous injection of 1-2 UI NPH insulin (Humulin N, Lilly, Fegersheim, France) for 8 weeks according to literature review [28]. Based on prior study, Group B received daily 300 mg/kg of berberine (Sigma, St. Louis, MO, USA) dissolved in 0.5% carboxymethyl cellulose via oral gavage [29]. Group IB received insulin and berberine with doses and methods similar to those of Groups I and B. Group HC received a daily gavage of saline solution. A summary of the animal grouping and treatments is presented in Figure 2.

### 2.5. Fluorochrome Double-Labeling

Two fluorochromes were injected sequentially: alizarin red (25 mg/kg/im, Sigma, St. Louis, MO, USA) at 1 week after surgery and calcein green (30 mg/kg/im, Sigma, St. Louis, MO, USA) at 7 weeks after surgery. Both fluorochrome labels could bind to the sites of active bone deposition shortly after injection. This enabled the identification of bone deposition around implants at different time points.

### 2.6. Measuring of Blood Glucose and Body Weight

Blood samples were randomly obtained from the animals by tail snipping, with plasma glucose levels (PGLs) measuring by an Accu-Chek glucose meter (Roche Diagnostics, Laval, QC, Canada). PGLs and body weights of the rats (*n* = 10 specimens per group) were measured at −3 weeks, 0 days, 1 week, 4 weeks, and 8 weeks, respectively.

### 2.7. Serum Biochemical Assays

All the animals were sacrificed 8 weeks after surgery with a lethal dose of pentobarbital. Blood samples (*n* = 10 specimens per group) were collected at the time of sacrifice and analyzed for serum alkaline phosphatase (ALP) and bone gamma-carboxyglutamic acid-containing (also known as osteocalcin) protein (BGP). Serum values of BGP were measured by rat osteocalcin Enzyme-Linked Immunosorbent Assay (ELISA) Kit (DRG International Inc., NJ, USA). ALP activity was analyzed using a pNPP Alkaline Phosphatase Assay Kit (AnaSpec Inc., CA, USA).

### 2.8. Fluorochrome Double-Labeling Analysis

The tibias with implants (*n* = 10 specimens per group) were harvested after the removal of soft tissue and maintained in a 4% neutral-buffered solution of formalin at 4°C for fixation. The specimens were dehydrated in an ascending series of alcohol concentrations and embedded with methyl methacrylate. Afterwards, the samples were sectioned along the longitudinal direction of the implant by a rotary diamond saw (SP1600/2600, Leica, Nussloch, Germany) and each tibia specimen could get 3 to 4 slides, which were ground to a final thickness of approximately 30 *μ*m by a microtome (Leica SP2600 Ultramiller, Nussloch, Germany). The slides were first observed under a Nikon Eclipse 300 fluorescence microscope (Compix Inc., Sewickley, PA, USA) to determine the mineral apposition at different time points.

### 2.9. Histomorphological Analysis

After the labeling analysis, the slides with section through the central part of screw in each tibia specimen were selected and stained with 1% toluidine blue (*n* = 10 slides per group). Images were captured through a microscope (DXM 1200, Nikon, Tokyo, Japan) and evaluated using a computerized image analyzer (Image Pro Plus V6.0, Media Cybernetics, Bethesda, MD, USA). Values of bone-to-implant contact ratio (BIC, the percentage of linear surface of the implant directly contacted by mineralized bone) were measured along the whole implant surface and bone area fraction occupancy (BAFO, the percentage of bone filling the spaces between implant threads) was evaluated in the spaces within the whole implant threads [30].

### 2.10. Statistical Analysis

All of the experiments were repeated three times, and means ± SDs (standard deviations) were calculated for each array of treatments for preliminary numerical comparisons. Intergroup differences were first analyzed using one-way ANOVA followed by Fisher's Least Significant Difference (LSD) post hoc tests at a significance level of *α* = 0.05. All data was analyzed with SPSS 19.0 software (SPSS, Inc., Chicago, USA).

## 3. Results

At 8 weeks postoperatively, all the surgery sites showed adequate healing, with no implant loss or infection.

### 3.1. Plasma Glucose Levels

Plasma glucose levels were presented in Table 1. Before STZ administration, the average of PGLs in each treatment group ranged from 5.8 to 6.3 mmol/L, considered to be normal in this study. After STZ administration, PGLs in the diabetic rats showed a significant increase. After treatment, both insulin and/or berberine effectively lowered the PGLs. At the end of the study, Groups I and IB had PGLs restored to their prestudy levels. Berberine moderately lowered PGLs to 11.2 mmol/L.

### 3.2. Body Weight

Table 2 shows the fluctuation of animal weights. After diet manipulation, animals fed with HFD weighed significantly greater than those fed with NPD (*P* < 0.05). However, the induction of diabetes caused a rapid loss of weights. At the end of study, DM rats in Groups I and IB gained weight; nevertheless, rats in Groups D and B continued to lose weight.

### 3.3. Plasma Levels of BGP and ALP

**Figure 3:**
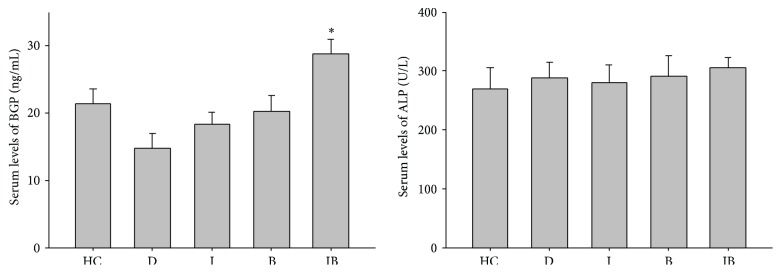
Plasma BGP and ALP levels at 8 weeks after implantation. ^*∗*^ *P* < 0.05, for Group HC versus Group IB.

Figure 3 showed that, after 8 weeks, insulin and/or berberine substantially increased the plasma BGP levels compared to that of Group D. Moreover, the plasma BGP levels in Group IB were significantly higher than that in Group HC (*P* < 0.05). As for the plasma ALP levels, the values in Groups B and IB seem to be higher than Group D; the differences were not significant (*P* > 0.05).

### 3.4. Fluorescence Observations

**Figure 4:**
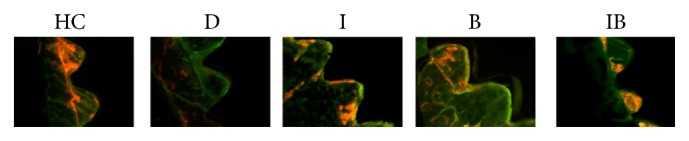
Fluorochrome double-labeling images of peri-implant osseointegration under microscopy at 10x (HC: healthy control; D: nontreated diabetic; I: insulin-treated diabetic; B: berberine-treated diabetic; IB: berberine + insulin-treated diabetic). Red was labeled by alizarin red at week 1 after surgery and green was labeled by calcein green at week 7 after surgery.

Figure 4 showed mineral appositions around the implant, which was determined by fluorochrome double-labeling. The areas in green or red represented regions of calcium precipitation labeled by fluorochromes during the healing phases: red being at week 1 and green at week 7 after surgery. All groups but the diabetic exhibited obvious red labeling: larger scales of red labeling were observed in Groups HC and IB, whereas only a few red spots were observed in Group D. In addition, only faint green labeling was observed in all groups.

### 3.5. Histomorphometric Analyses

**Figure 5:**
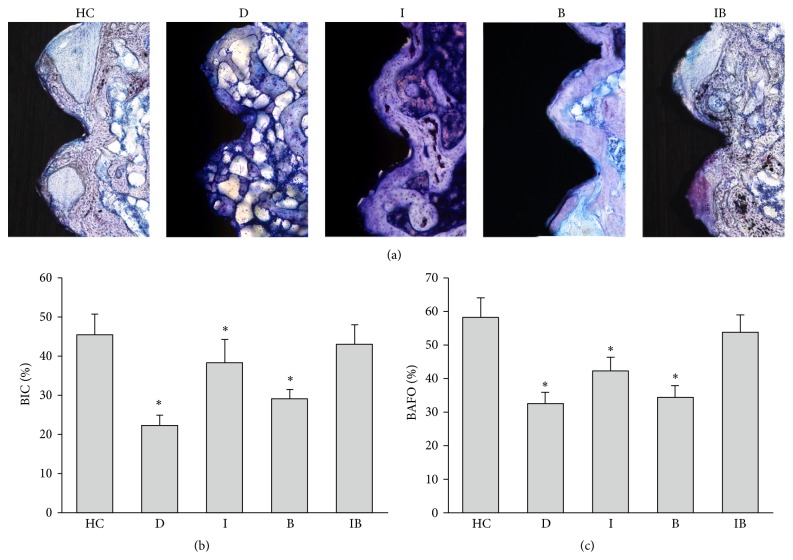
Histological images and quantitative analysis of implants 8 weeks after surgery by toluidine blue staining (10x). (a) HC: healthy control; D: nontreated diabetic; I: insulin-treated diabetic; B: berberine-treated diabetic; IB: berberine + insulin-treated diabetic. (b) Bone-to-implant contact ratio. (c) Bone area fraction occupancy ratio. Data are expressed as mean ± SD, *n* = 10. ^*∗*^ *P* < 0.05, for healthy control rats versus others.

Figure 5(a) showed bone formation around implants. Comparison of bone filling the spaces between threads: there was the least osseointegration (discontinuance bone, minor direct contact) in Group D and the most osseointegration (mostly lamellar bone, dense and well organized, major contact) in Groups HC and IB. In quantitative analysis (Figures 5(b) and 5(c)), insulin increased BIC 1.7-fold and BAFO 1.3-fold compared with Group D. Berberine increased BIC 1.3-fold and slightly increased BAFO. The BIC and BAFO were successfully restored to normal level after insulin and berberine combined treatment (no significant differences compared with those of Group HC (*P* > 0.05)).

## 4. Discussion

Diabetes mellitus has been widely studied for its influence on bone healing and remodeling and is shown to be detrimental to dental implants osseointegration. Consistent with previous studies [11], impaired new bone formation (little fluorochrome labeling) and poor osseointegration (decreased BIC and BAFO values) were observed in the untreated diabetic rats.

Previous studies have mainly discussed application of insulin to control serum glucose and improve implant osseointegration. However, the effects remained controversial due to the heterogeneity of diabetic models and the methodologies of insulin therapy used in their studies. The diabetic rat model in this study was induced by two steps: first, high-fat chow feeding to induce obesity, compensatory hyperinsulinemia, and insulin resistance, and second, the administration of low-dose STZ to destroy a part of pancreatic beta cells, leading to a reduction of insulin in serum and causing hyperglycemia. This STZ model is a widely used model in type 2 diabetic research that replicates metabolic characteristics of the human syndrome [26]. In this study, the changes of plasma glucose levels and weight substantiated onset of diabetic symptoms. The rats in Group D had significantly higher PGLs and increased body weight loss. The diabetic animals treated by insulin had their PGLs gradually restored to the normal range, indicating continuous regulation of plasma glucose levels in this study, which would avoid the major side effect of hypoglycemia usually seen in an intensive insulin therapy [31]. In fact, the fear of hypoglycemia commonly complicated the effective glycemic control both in patients and in physicians [32]. In this study, insulin only moderately increased BIC and BAFO, which were not comparable to the healthy controls. This might contribute to the fact that the body cells could not use insulin effectively due to insulin resistance (defects in insulin-related signaling). Our study showed that insulin could not counterbalance all the negative changes caused by diabetes, which was in agreement with former studies showing that the BIC and removal torque were significantly lower compared to the healthy controls [9, 10].

Berberine has been recently reported for its dual functions in boosting bone remodeling and maintaining glucose homeostasis. Consistent with previous observations, the administration of berberine alone could effectively lower PGL but failed to restore it to the normal level [18]. The explanation might be that although berberine could activate both insulin and AMPK signaling to increase glucose uptake, it could not alter the insulin secretion and synthesis therefore did not reverse total insulin reduction caused by destroyed pancreas beta cells [33]. This incomplete control of hyperglycemia may further explain weaker promotion of berberine on osseointegration compared with insulin. Interestingly, no weight gains were observed in Group B though PGLs were reduced, implying that berberine might have weaker or delayed regulation on body energy homeostasis compared to its effect on glucose modulation.

We further investigated the effect of berberine and insulin combined treatment on implant osseointegration and whether the effect is additive. As is shown in PGLs result, berberine together with insulin treatment successfully corrected PGLs after 4 weeks. Furthermore, values of BIC and BAFO in Group IB increased and were equivalent to those observed in Group HC. As ALP and BGP secreted by osteoblasts are associated with early and subsequent bone formation stages [34, 35], serum measurement of these two biomarkers in correlation with histological assessments of osseointegration can provide important information about the influence of the treatment on bone metabolism. The serum total ALP level in Groups B and IB was higher than that of Group D, while the difference was not significant (*P* > 0.05). Interestingly, the correlation between serum ALP levels and localized osseointegration was not reflected. This might be due to the fact that alkaline phosphates are secreted by various sources including nonskeletal tissues, such as liver, intestine, and spleen. We also found significantly increased serum BGP levels in Groups I, B, and IB, with the highest value in Group IB. Taken together, the corrected PGLs, improved osseointegration, and highest levels of BGP in Group IB suggest that berberine in combination with insulin may lead to an additive or synergistic effect on glycemic control and bone activity. The improved osseointegration might be attributed to multiple factors mainly including corrected hyperglycemia, enhanced bone formation, and inhibited bone resorption: (1) abnormality in insulin receptor (InsR) is the major cause for the development of insulin resistance and type 2 diabetes mellitus. Berberine mimics insulin action and attenuates insulin resistance by increasing IR phosphorylation [15]. Therefore, when applied together, berberine and insulin may have additive effect in glucose uptake. (2) Berberine has potent antioxidant effects in reducing protein damage, DNA stand breaking, and inflammatory markers level in diabetic rats, which is beneficial for maintaining a suitable environment for bone healing [36, 37]. (3) Berberine decreases the level of bone loss by inhibiting osteoclasts formation and differentiation [22].

With the steady increase of diabetes patients, therapies that promote higher clinical efficiency and shorter rehabilitation duration are of great urgency in dental implant treatment. This study took advantage of the multiple functions of berberine in glucose homeostasis, insulin sensitizing, and bone anabolism, which are closely related to the promotion of bone formation in diabetic patients. We found that a combination of insulin and berberine (two existing drugs) seemed to be more effective than each applied alone, which may be because of their different and complementary mechanisms of action. Admittedly, differences of development and characterization could exist between the tibia and jaw. Further analysis at the cellular and molecular levels is also helpful to elucidate the intrinsic mechanism of action of the combination therapy. Also, modifying the chemistry of berberine to enhance its properties in glucose homeostasis and bone anabolism may also be encouraged.

## Figures and Tables

**Table 1:** Plasma glucose levels (mmol/L) of rats during the whole study.

Animal status	−3 weeks	0 days	1 week	4 weeks	8 weeks
Group HC	5.8 ± 0.7^1^	6.4 ± 0.8^1^	6.3 ± 0.8^1^	6.6 ± 0.8^1^	6.7 ± 0.7^1^
Group D	6.2 ± 0.7^1^	27.1 ± 2.2^2^	24.0 ± 2.4^5^	29.1 ± 1.9^3^	25.4 ± 1.2^3^
Group I	6.1 ± 0.8^1^	26.8 ± 3.0^2^	11.2 ± 1.8^3^	8.7 ± 1.2^1^	7.1 ± 0.6^1^
Group B	6.2 ± 0.7^1^	26.8 ± 3.7^2^	20.1 ± 1.6^4^	17.5 ± 2.2^2^	11.2 ± 1.3^2^
Group IB	6.3 ± 0.6^1^	24.4 ± 1.4^2^	8.7 ± 1.7^2^	6.4 ± 1.2^1^	6.7 ± 0.9^1^

Means with identical superscripts in a given column are not statistically significant at the 5% alpha level.

**Table 2:** Weights (g) of rats during the whole study.

Animal status	−3 weeks	0 days	1 week	4 weeks	8 weeks
Group HC	207 ± 2.8^1^	262 ± 21.9^1^	275 ± 20.3^1^	344 ± 19.7^2^	424 ± 20.0^3^
Group D	212 ± 2.9^1^	314 ± 15.4^2^	291 ± 13.8^1^	289 ± 18.4^1^	267 ± 18.1^1^
Group I	209 ± 3.9^1^	323 ± 30.1^2^	301 ± 19.5^1^	308 ± 23.4^2^	374 ± 30.6^2^
Group B	212 ± 5.3^1^	314 ± 29.4^2^	302 ± 25.4^1^	284 ± 28.9^1^	277 ± 27.7^1^
Group IB	210 ± 3.9^1^	317 ± 20.7^2^	307 ± 26.8^1^	313 ± 30.0^2^	397 ± 27.9^2^

Means with identical superscripts in a given column are not statistically significant at the 5% alpha level.
